# The influence of atorvastatin on parameters of inflammation left ventricular function, hospitalizations and mortality in patients with dilated cardiomyopathy – 5-year follow-up

**DOI:** 10.1186/1476-511X-12-47

**Published:** 2013-04-08

**Authors:** Agata Bielecka-Dabrowa, Dimitri P Mikhailidis, Manfredi Rizzo, Stephan von Haehling, Jacek Rysz, Maciej Banach

**Affiliations:** 1 Department of Hypertension, Chair of Nephrology and Hypertension, Medical University of Lodz, Lodz, Poland; 2 Department of Clinical Biochemistry, Royal Free Campus, University College London Medical School, University College London (UCL), London, UK; 3 BioMedical Department of Internal Medicine and Medical Specialties, University of Palermo, Palermo, Italy; 4 Euro-Mediterranean Institute of Science and Technology, Palermo, Italy; 5 Applied Cachexia Research, Department of Cardiology, Charité Medical School, Campus Virchow-Klinikum, Berlin, Germany; 6 Department of Nephrology, Hypertension and Family Medicine, Chair of Nephrology and Hypertension, Medical University of Lodz, Lodz, Poland

**Keywords:** Atorvastatin, Dilated cardiomyopathy, Heart failure, Inflammation

## Abstract

**Background:**

We assessed the influence of atorvastatin on selected indicators of an inflammatory condition, left ventricular function, hospitalizations and mortality in patients with dilated cardiomyopathy (DCM).

**Methods:**

We included 68 DCM patients with left ventricular ejection fraction (LVEF) ≤40% treated optimally in a prospective, randomized study. They were observed for 5 years. Patients were divided into two groups: patients who were commenced on atorvastatin 40 mg daily for two months followed by an individually matched dose of 10 or 20 mg/day (group A), and patients who were treated according to current recommendations without statin therapy (group B).

**Results:**

After 5-year follow-up we assessed 45 patients of mean age 59 ± 11 years - 22 patients in group A (77% male) and 23 patients in group B (82% male). Interleukin-6, tumor necrosis factor alpha, and uric acid concentrations were significantly lower in the statin group than in group B (14.96 ± 4.76 *vs.* 19.02 ± 3.94 pg/ml, *p* = 0.012; 19.10 ± 6.39 *vs.* 27.53 ± 7.39 pg/ml, *p* = 0.001, and 5.28 ± 0.48 *vs.* 6.53 ± 0.46 mg/dl, *p* = 0.001, respectively). In patients on statin therapy a reduction of N-terminal pro-brain natriuretic peptide concentration (from 1425.28 ± 1264.48 to 1098.01 ± 1483.86 pg/ml, *p* = 0.045), decrease in left ventricular diastolic (from 7.15 ± 0.90 to 6.67 ± 0.88 cm, *p* = 0.001) and systolic diameters (from 5.87 ± 0.92 to 5.17 ± 0.97, *p* = 0.001) in comparison to initial values were observed. We also showed the significant increase of LVEF in patients after statin therapy (from 32.0 ± 6.4 to 38.8 ± 8.8%, *p* = 0.016). Based on a comparison of curves using the log-rank test, the probability of survival to 5 years was significantly higher in patients receiving statins (*p* = 0.005).

**Conclusions:**

Atorvastatin in a small dose significantly reduce levels of inflammatory cytokines and uric acid, improve hemodynamic parameters and improve 5-year survival in patients with DCM.

## Introduction

According to the European Society of Cardiology (ESC), dilated cardiomyopathy (DCM) is recognized based on dilation and systolic dysfunction of the left ventricle, unless a patient simultaneously suffers from coronary artery disease (CAD), hypertension, valvular heart disease or congenital heart disease which is so significant that it leads to an observed pathology of the myocardium [1].

DCM occurs more frequently in men and is most common between the ages of 20 and 60 years [1,2]. About 1 in 3 cases of congestive heart failure (CHF) is due to DCM. Despite treatment, CHF is a progressive disease with high morbidity and mortality, suggesting that important pathogenic mechanisms remain active and unmodified by currently available treatment [1,2].

The cause of mortality in these patients is either end-organ dysfunction due to pump failure or arrhythmia-related death [3,4]. Within recent years more and more evidence has been presented indicating that autoimmunologic processes, cellular as well as humoral ones, are involved in the pathogenesis of dilated cardiomyopathy [3]. The common presence of viral genetic material in the myocardium of patients with DCM provides the most compelling evidence, but proof of causality is still lacking [3,4]. In addition, autoimmune reactions have been described in many studies, indicating them as an important etiologic factor [3,4]. A pivotal role for autoimmunity in a substantial proportion of patients with DCM is supported by the presence of organ-specific autoantibodies, inflammatory infiltrates and pro-inflammatory cytotoxic cytokines [3-6]. Furthermore, familial occurrence of DCM has been described in about 20–30% of cases, with the presence of autoantibodies and abnormal cytokine profiles in first-degree relatives with asymptomatic left ventricular enlargement [3,4]. This suggests the involvement of a disrupted humoral and cellular immunity early in the development of the disease [3,4].

Despite their lipid-lowering and anti-atherosclerotic effects statins have also important pleiotropic properties. They limit signal transmission from membrane receptors and slow down pathologic remodeling of the heart and vessels, inhibit the action of angiotensin II, inhibit apoptosis, improve endothelial function, retard the progression of heart failure symptoms, reduce the level of N*-*terminal pro-brain natriuretic peptide (NT-proBNP), restore autonomic nervous system balance and might have a protective influence on renal function and blood pressure [7,8]. Previous studies have demonstrated that statins reduce vascular and myocardial oxidative stress by inhibiting *Rac*-induced nicotinamide adenine dinucleotide phosphatase (NADPH) oxidase activity and reducing oxidized low density lipoprotein (LDL) concentration by activated monocyte-derived macrophages [9]. They also reduce intracellular reactive oxygen species (ROS) in endothelial cells by S-nitrosylation of thioredoxin [9,10].

The Rosuvastatin Impact on Ventricular Remodelling Lipids and Cytokines (UNIVERSE), Controlled Rosuvastatin Multinational Trial in Heart Failure (CORONA), and Effect of rosuvastatin in patients with chronic heart failure (GISSI-HF) trials did not indicate a significant role for statins in HF patients, although the drug did reduce the number of cardiovascular (CV) hospitalizations in the CORONA trial [11-13]. Although mentioned prospective studies using hydrophilic rosuvastatin showed no beneficial effect on mortality, Vrtovec *et al.* reported that atorvastatin therapy reduced the incidence of sudden cardiac death in patients with advanced CHF [14]. Correale *et al.* evaluated the effect of statin therapy on left ventricular dysfunction in patients with CHF and showed that they had fewer readmissions for adverse events, blunted inflammatory activation and improved left ventricular performance assessed by tissue Doppler imaging [15]. Metabolic and cardiac effects may differ between the lipophilic and hydrophilic statins [16].

Therefore the aim of our study was to assess the association between 5-year atorvastatin therapy and indicators of an inflammatory condition and clinical outcomes in patients with DCM.

## Methods

### Study population

In a prospective study, 68 patients with DCM (according to the 2008 *European Society of Cardiology* [ESC] classification) of either sex, aged 18 years or older with left ventricular ejection fraction (LVEF) ≤40% treated with optimal medical therapy, were followed for 5 years [17,18]. Mean disease duration was 7.5 ± 1.9 years. No patients had significant coronary artery disease (>30% stenosis) as determined by cardiac catheterization performed within a year before the enrolment [19,20]. Arterial hypertension was not diagnosed in any of the patients. Date of death was ascertained by questioning relatives or patients’ general practitioners and estimated as close as possible to half year frames. Patients were randomized to one of two groups: A – patients who were commenced on atorvastatin 40 mg daily for 2 months followed by an individually matched dose of 10 or 20 mg/day; and B – patients who were treated according to current guidelines [21] without statin therapy.

The exclusion criteria were as follows: blood pressure (BP) ≥140/90 or <90/60 mmHg; congenital heart disease; acquired valvular disease with the exception of mitral incompetence secondary to left ventricular dilatation; persistent hyperactivity of aminotransferases with an unexplained etiology; muscle disorders which might cause drug-induced myopathy; uncontrolled diabetes; liver diseases, creatinine level >2 mg/dl and/or glomerular filtration rate (GFR) <30 ml/min; alcohol or drug abuse; chronic inflammatory diseases, pregnancy or lactation, severe hypothyroidism, immunosuppressive treatment, operation or severe injury during the month prior to blood collection, and patients who did not provide written informed consent.

Initial and control tests included full clinical examination with the assessment of body mass index (BMI) and New York Heart Association (NYHA) class, routine laboratory tests, measurement of TNF-α, interleukin 6 (IL-6), and transforming growth factor beta (TGF-β) concentrations in blood plasma, measurement of N-terminal pro-brain natriuretic peptide (NT-proBNP), syndecan-4, cystatin C (CysC) concentration in blood serum, echocardiographic examination and the assessment of exercise capacity in 6-min walk test (6MWT). The frequency of HF hospitalization and mortality were recorded during the 5-year follow-up period.

Consent from the Bioethics Commission of the Medical University of Lodz (No. RNN/520/10/KB) was obtained. Written informed consent was obtained from all the patients.

### Biochemical tests

Blood glucose was measured with a glucose dehydrogenase method after precipitation of proteins by trichloroacetic acid. LDL and high-density lipoprotein (HDL) fractions were separated from fresh serum by combined ultracentrifugation and precipitation. Lipoprotein fraction cholesterol and triglycerides were measured enzymatically. The concentration of NT-proBNP was determined using an Elecsys 2010 analyzer (Roche Diagnostics, Warsaw, Poland). After the blood was taken, the material was centrifuged; the obtained serum was frozen at the temperature of −70°C and stored in this condition until the time of examination.

The determination of NT-proBNP in blood serum was performed with the electroluminescence method with 2 polyclonal antibodies directed against NT-proBNP within epitope 1 (1–21 amino acid sequence) and epitope 2 (39–50 amino acids). Concentration values are given in pg/ml. Determination of IL-6 and TNF-α was performed with reagents of Beckman Coulter (Paris, France), using a sandwich ELISA assay. Measurement of CysC was performed using immunonephelometric assay for the quantitative measurement of this marker in human serum and heparinized plasma. Diazyme’s Cystatin C assay is based on the latex-enhanced immunoturbidimetric method. The range of valid values for CysC measured by the immunonephelometric method (N Latex Cystatin C test) is 0.53–0.95 mg/l. Determination of syndecan 4 was performed with reagents of USCN Life Science Inc. (Wuhan, China),using a sandwich ELISA assay [19]. Determination of TGF-β was performed with reagents of Diaclone/Gen-Probe (San Diego, USA), using an enzyme-linked immunosorbent assay.

### Echocardiographic assessment

Echocardiography was performed using an ALOKA Alpha 6 Premier (Tokyo, Japan) with a 3–11 MHz probe. Left ventricular (LV) systolic function and cardiac dimensions indexed to body surface area were determined. The heart was imaged in parasternal short axis view to obtain LV wall thickness and parasternal long axis view to measure ejection fraction (EF), which was determined with Simpson’s rule: *EF = (LVEDV-LVESV)/LVEDV*, where LVEDV is left ventricular end-diastolic volume and LVESV is left ventricular end-systolic volume.

Left ventricular end-diastolic diameter (LVEDD) and left ventricular end-systolic diameter (LVESD) were measured from M-mode tracings. Flow parameters were assessed in Doppler examination (continuous, pulsatile and color). Quantification of LV systolic function was also made through application of:

A. *The myocardial performance index* (MPI), which reflects global efficiency of LV functioning. It is determined by dividing the sum of isovolumetric relaxation time and isovolumetric contraction time by ejection time. The time of isovolumetric contraction is measured from the closure of the mitral valve to the opening of the aortic valve. The time of isovolumetric relaxation is measured from the closure of the aortic valve to the opening of the mitral valve. The norm is ≤0.4; higher values indicate deteriorating efficiency of the myocardium [20].

B. *Rate of systolic pressure change in the left ventricle* (dP/dT). This index determines the increase in systolic pressure generated by the LV in time calculated using continuous wave (CW) Doppler based on the time of increase in the speed of mitral regurgitation (MR) from 1 to 3 m/s. Values of dP/dT <400 mmHg/s indicate that the systolic function of the LV is significantly impaired; normal values are >2000 mmHg/s [20,22].

Diastolic function of the LV was assessed using the parameters of mitral inflow registered with pulsed wave (PW) Doppler in 4-cavity apical projection and diastolic speed values of movement of the mitral ring registered with tissue Doppler imaging.

### Statistical analysis

The STATISTICA software package 9 (StatSoft, Poland) was used for analysis. All values presented are the mean or median ± standard deviation (SD) for continuous variables and the percentage of total patients for categorical variables. The Shapiro-Wilk test was used to assess the normality of distribution. To compare groups Student’s *t* test or 2-way analysis of variance (ANOVA) for continuous and discrete variables with normal distribution and non-parametric Mann-Whitney *U* test if the distribution was not normal were applied. For categorical variables chi-square test or Fisher’s test for small samples was applied for comparisons. For quantitative variables (continuous and discrete) to check correlations between variables Spearman’s rank correlation coefficient was used. The assessment of factors influencing prognosis in patients with DCM was performed using single-factor logistic regression and the forward stepwise regression model, and receiver operating characteristic (ROC) curves and the forward stepwise regression model. Based on analysis of the ROC curve, cut-off points were found for the measurable variables by estimating the highest product of sensitivity and specificity. Calculations and drawings were made using SPSS 17.0 statistical package. The analysis of death was plotted using the Kaplan-Meier method. Date of death was ascertained by questioning relatives or patients’ general practitioners and estimated as close as possible to half year frames. Results were considered significant at p < 0.05.

## Results

### Baseline characteristics of the patients

During 5-year observation 29% (n = 20) of all 68 patients died, 41% (n = 28) required one re-hospitalization and 6% (n = 4) two re-hospitalizations because of HF decompensation. In 9% (n = 6) cardiac resynchronization therapy was applied, mitral and tricuspid valvuloplasty was performed in 1 person and heart transplantation in 2 people (these patients were excluded from the analysis to avoid the imposition of the effect of statins and the results of such procedures).

Finally after 5 years we assessed 45 patients of mean age 59 ± 11 years (5 females and 40 males): group A– 22 patients (77% male) of mean age 63 ± 10 years treated with atorvastatin in individual dose of 10 (n = 11) or 20 mg (n = 11), and group B – 23 patients (82% male) of mean age 57 ± 13 years without statin therapy. In 36% (n = 16) of patients we observed dyspnea, pulmonary hemostasis in 11% (n = 5), and edema in 9% (n = 4). Body mass assessment revealed underweight in 4% (n = 2) of patients, normal weight in 36% (n = 16), overweight in 24% (n = 11) and obesity in 36% (n = 16). Chronic obstructive pulmonary disease was present in 4% (n = 2) and diabetes mellitus (DM) or abnormal glucose level in 20% (n = 9) of patients. The detailed characteristics of the patients are presented in Table 1.

**Table 1:** Characteristics of the analyzed patients with DCM after 5 years

**PARAMETER**	**Mean ± SD or number of patients (%)**
Age (years)	59.0 ± 11.0
Gender (male)	40 (89)
NYHA class	
I	4 (9)
II	22 (49)
III	15 (33)
IV	4 (9)
Atrial fibrillation	15 (33)
Hypercholesterolemia	20 (46)
Anemia*	4 (9)
Diabetes mellitus or abnormal glucose level	9 (20)
LV diastolic dysfunction	4 (9)
sPAP >40 mmHg	4 (9)
MR degree	
0	7 (15)
I	19 (43)
II	15 (34)
III	4 (9)
TR degree	
0	23 (51)
I	14 (32)
II	8 (17)
**PHARMACOLOGICAL TREATMENT**	
Loop diuretics	41 (91)
Spironolactone/eplerenone	39 (87)
vBeta-blockers	40 (88)
ACE inhibitors	41 (91)
Sartans (ARBs)	4 (9)
Digoxin	15 (34)
Acenocoumarol/warfarin	9 (20)
Amiodarone	4 (8)
Statins	22 (49)
Insulin	2 (4)
Oral hypoglycemics	4 (9)

*Anemia: Hb (g/dL) <13 in men and <12 in women.

**ABBREVIATIONS**: *ACE* inhibitors – angiotensin converting enzyme inhibitors; *ARB* angiotensin receptor blocker; *MR* mitral regurgitation; *NYHA* New York heart association; *sPAP* systolic pulmonary artery pressure; *TR* tricuspid regurgitation.

### The influence atorvastatin treatment on inflammatory and clinical parameters

In group A compared to group B, IL-6 concentration was considerably lower (14.96 ± 4.76 *vs* 19.02 ± 3.94 pg/ml, *p* = 0.011) after five years of treatment with atorvastatin. TNF-α levels were also significantly reduced in the statin group than in Group B (19.10 ± 6.39 *vs* 27.53 ± 7.39 pg/ml, *p* = 0.001). Also uric acid (UA) concentration was lower in the atorvastatin group (5.28 ± 0.48 *vs* 6.53 ± 0.46 mg/dl, *p* = 0.001). No significant differences concerning NT-proBNP concentration, echocardiographic parameters of the left ventricle, distance on 6MWT and in functional classification according to NYHA were observed between examined groups. The detailed comparison between groups A and B is presented in Table 2.

**Table 2:** Comparison of selected parameters in patients with DCM from group A (atorvastatin group) and group B (without statin) after 5-year follow-up

**PARAMETER**	**Group A (n = 22)**	**Group B (n = 23)**	***p***
	**Mean ± SD**	**Mean ± SD**	
BMI	28.9 ± 4.3	31.0 ± 4.4	ns*
Waist circumference (cm)	98.3 ± 12.9	96.9 ± 13.5	ns
Systolic RR (mm Hg)	114.7 ± 13.0	117.5 ± 7.7	ns
Diastolic RR (mm Hg)	71.5 ± 7.7	77.3 ± 5.3	ns
Heart rate	77 ± 9	69 ± 4	ns
***Biochemical parameters***			
White blood cells (*10^3^/μl)	6.9 ± 1.4	7.3 ± 1.4	ns
Hemoglobin (g/dl)	14.6 ± 1.4	14.5 ± 1.3	ns
Na (mmol/l)	138 ± 4	138 ± 3	ns
K (mmol/l)	4.4 ± 0.4	4.5 ± 0.5	ns
Total cholesterol (mg/dl)	178 ± 69	202 ± 30	***0.02***
HDL cholesterol (mg/dl)	52 ± 18	48 ± 9	ns
LDL cholesterol (mg/dl)	78 ± 25	123 ± 27	***0.001***
Triglycerides (mg/dl)	201 ± 212	153 ± 37	ns
ASPAT (U/l)	23.6 ± 6.3	26.2 ± 9.6	ns
ALAT (U/l)	23.5 ± 7.4	26.3 ± 10.7	ns
Urea (mg/dl)	30.80 ± 14.00	39.33 ± 7.84	ns
Creatinine (mg/dl)	0.92 ± 0.27	1.00 ± 0.32	ns
Uric acid (mg/dl)	5.28 ± 0.48	6.53 ± 0.46	***0.001***
CRP (mg/l)	1.4 ± 1.1	2.7 ± 2.5	ns
GRF MDRD (ml/min)	792 ± 22	71 ± 19	ns
***Biomarkers***			
NT-proBNP (pg/ml)	1098.00 ± 1483.86	1151.24 ± 1371.00	ns
TNF-alpha (pg/ml)	19.10 ± 6.40	27.53 ± 7.39	***0.001***
Interleukin 6 (pg/ml)	14.90 ± 4.70	19.00 ± 3.94	***0.01***
Syndecan 4 (ng/ml)	4.40 ± 3.10	5.21 ± 4.00	ns
Cystatin C (mg/l)	1.17 ± 0.39	1.00 ± 0.18	ns
TGF-beta (pg/ml)	470.19 ± 166.50	463.00 ± 156.78	ns
***Echocardiographic parameters***			
LVdD (cm)	6.6 ± 0.8	6.4 ± 1.8	ns
LVsD (cm)	5.1 ± 0.9	5.1 ± 1	ns
LVEF (%)	38.4 ± 8.8	33.9 ± 11.5	ns
LA diameter (cm)	4.7 ± 0.8	8.0 ± 10.5	ns
RVdD(cm)	2.9 ± 0.4	3.0 ± 0.6	ns
LVEdV (ml)	187.0 ± 37.0	191.6 ± 72.9	ns
LVsV(ml)	118.9 ± 41.0	104.0 ± 41.5	ns
TEI index	0.7 ± 0.2	0.5 ± 0.4	ns
Mitral regurgitation dP/dT (mmHg/s)	569.5 ± 294.1	392.5 ± 83.0	ns
***Clinical state of patients***			
6-MWT (m)	419.3 ± 116.0	376.7 ± 114.3	ns
NYHA class (No. of patients [%])			
I	1 (5)	3 (13)	ns
II	12 (54)	10 (44)	
III	8 (36)	7 (30)	
IV	1 (5)	3 (13)	

* ns – not statistically significant.

**ABBREVIATIONS:***6 MWT* 6-minute walk test; *BMI* body mass index; *Na* sodium; *K* potassium; *HDL* high-density lipoprotein; *LDL* low-density lipoprotein; *ASPAT* aspartate transaminase; *ALAT* alanine transaminase; *CRP* C-reactive protein; *NT-proBNP* N-terminal pro-brain natriuretic peptide; *TNF* tumor necrosis factor; *TGF* beta*–*transforming growth factor beta; *GFR MDRD* glomerular filtration rate using modification of diet in renal disease formula; *LVdD* left ventricular diastolic diameter; *LVsD* left ventricular systolic diameter; *LVEF* left ventricular ejection fraction; *LA* left atrium; *RVdD* right ventricular diastolic diameter; *LVEdV* left ventricular end-diastolic volume; *LVsV* left ventricular systolic volume; *NYHA*; New York heart association.

### Patients with and without atorvastatin therapy – comparing changes over time

In the statin group after 5 years a decrease in NT-proBNP concentration compared with initial values from 1425.28 ± 1264.48 to 1098.01 ± 1483.86 pg/ml (*p* = 0.045) and a decrease in LVdD and LVsD from 7.15 ± 0.90 to 6.67 ± 0.88 cm (*p* = 0.001) and from 5.87 ± 0.92 to 5.17 ± 0.97 (*p* = 0.001), respectively, were achieved. The significant increase of LVEF from 32.0 ± 6.4 to 38.8 ± 8.8% (*p* = 0.0164) was also observed in Group A. There were no significant changes in the range of these parameters in Group B. Only in the atorvastatin group a significant reductions of total cholesterol (from 207 ± 71 to 183 ± 69 mg/dL) and LDL cholesterol (from 116 ± 36 to 80 ± 25 mg/dL) were observed.

In the control group a significant increase in TNF-α levels from 12.70 ± 12.78 to 27.50 ± 7.39 pg/ml (*p* = 0.006) and an increase in body mass index (BMI) from 29.6 ± 4.5 to 31.0 ± 4.4 (*p* = 0.033) were found.

### Safety and tolerance of atorvastatin therapy

No patients had symptoms of myopathy. There were no significant changes in aminotransferases activities between investigated groups of patients.

### Hospitalization and survival assessment

The following factors influenced the risk of HF hospitalizations on the basis of single-factor logistic regression: leg edema, hepatomegaly, no beta-blocker therapy, NT-proBNP, LVEF, LVsD and result of the 6-MWT (Table 3). On the basis of single-factor logistic regression statistical analysis we found that: no statin therapy, leg edema, hepatomegaly, no beta-blocker therapy, renal failure, re-hospitalizations, lower BMI, LVEF, higher NT-proBNP, LVsD, LVdD, MR degree, significantly influenced the risk of death (Table 4).

**Table 3:** Predictors of re-hospitalizations in single-factor logistic regression

**Categorical variable**	**With re-hospitalization (%)**	**Without re-hospitalization (%)**	***p***
leg edema	91	9	0.009
hepatomegaly	88	11	0.03
No beta-blocker therapy	98	0	0.006
**Quantitative variable**	**With re-hospitalization (median; Q25–Q75)**	**Without re-hospitalization (median; Q25–Q75)**	***p***
BMI 2*	25; 24–30	30; 26–32	0.04
NT-proBNP 1	1848; 931–3248	895; 511–2026	0.02
NT-proBNP 2	1852; 775–4000	745; 360–1680	0.005
LVEF 1	27; 22–31	33; 28–38	0.02
LVEF 2	30; 24–32	34; 29–40	0.02
LVsD 2	6; 5.2–6.4	5.3; 4.4–5.6	0.001
MR 1	1.5; 1–2	1; 0–1	0.03
MR 2	1; 1–2	1; 0–1	0.03
6-MWT 1	350; 290–405	438; 350–490	0.01
6-MWT 2	370; 290–450	480; 395–595	0.001

*1 – for variables assessed after inclusion; 2 – for variables assessed after 2 months.

Q25–Q75: quartiles 25% and 75%.

**ABBREVIATIONS:***BMI* body mass index; *NT-proBNP* N-terminal pro-brain natriuretic peptide; *LVEF* left ventricular ejection fraction; *LVsD* left ventricular systolic diameter; *MR* mitral regurgitation; *6 MWT* 6 minute walk test.

**Table 4:** Predictors of death in single-factor logistic regression

**Categorical variable**	**Death (%)**	**Survival (%)**	***p***
No statin treatment	78	21	0.006
Leg edema	72	27	0.027
Hepatomegaly	66	33	0.009
No beta-blocker therapy	87.5	12.5	0.001
Renal failure	80	20	0.025
Re-hospitalizations	81	18	0.001
**Quantitative variable**	**With death (median; Q25–Q75)**	**Without death (median; Q25–Q75)**	***p***
BMI 1	24; 24–26	30; 25–32	0.002
BMI 2	24; 23–26	30; 25–32	0.003
NT-proBNP 1*	2771; 1500–5013	980; 500–2130	0.001
NT-proBNP 2	2900; 1296–4421	766; 470–1680	0.001
LVEF 1	27; 21–29	32; 26–38	0.01
LVEF 2	30; 22–31	32; 28–40	0.03
LVsD 2	6.2; 5.3–6.7	5.3; 4.5–5.9	0.01
LVdD 2	7.6; 6.7–8.4	6.6; 6.0–7.2	0.003
MR 1	2; 1–3	1; 0–1	0.001
MR 2	2; 1–3	1; 0–1	0.001

*1 – for variables assessed after inclusion; 2 – for variables assessed after 2 months.

Q25–Q75: quartiles 25% and 75%.

**ABBREVIATIONS:***BMI* body mass index; *NT-proBNP* N-terminal pro-brain natriuretic peptide; *LVEF* left ventricular ejection fraction; *LVsD* left ventricular systolic diameter; *LVdD* left ventricular diastolic diameter; *MR* mitral regurgitation.

**Figure 1:**
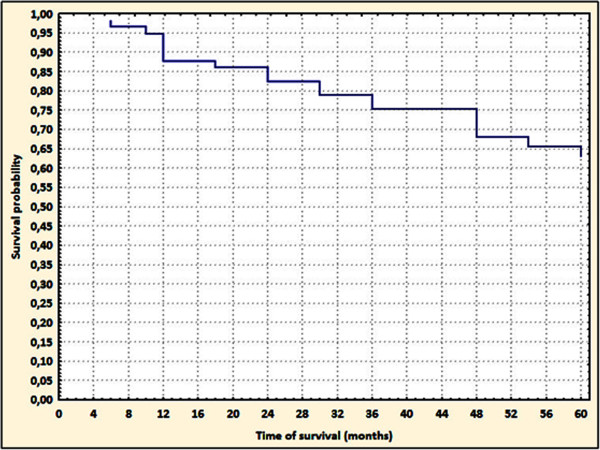
**Kaplan-Meier curves probability of survival in patients with DCM after 5-year follow-up.** *DCM – dilated cardiomyopathy.

**Figure 2:**
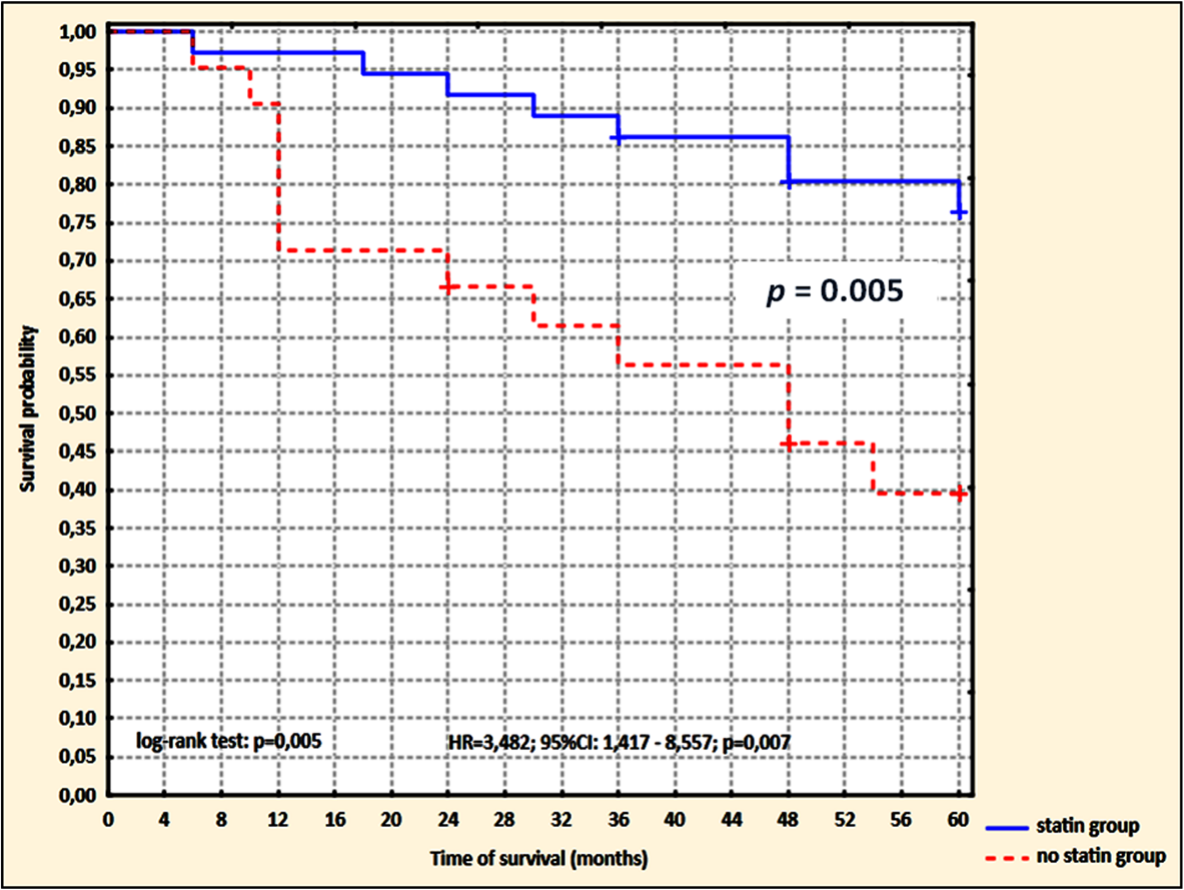
Probability of 5-year survival in patients with DCM treated and not treated with atorvastatin using Kaplan-Meier method.

The survival of patients was 87.7% (95%CI: 79.2–96.2%), 67.9% (95%CI: 55.7–80.2%), and 63.1% (95%CI: 50.0–76.2%) after 1, 2, and 5 years, respectively (Figure 1). Based on a comparison of curves using the log-rank test, the probability of survival to 5 years was significantly higher in the group taking a low dose of statin (*p* = 0.005; hazard ratio [HR] 3.48; 95%Cl 1.42–8.56) (Figure 2).

According to the multivariate regression analysis we found that NT-proBNP (odds ratio [OR] 1.00, *p* = 0.007) and LVdD (OR 2.96, *p* = 0.025) were the independent risk factors of death, and 6-MWT (OR 0.99, *p* = 0.001) was the only independent risk factors of re-hospitalization for worsening heart failure. On the basis of ROC curve analysis we found that NT-proBNP values ≥1826 pg/ml, LVEF ≤30%, BMI ≤25.5, and MR ≥1.5 were significant predictors of both re-hospitalization and increased mortality in this group of patients.

## Discussion

Statins act by inhibiting the enzyme 3-hydroxy-3-methylglutaryl coenzyme A (HMG-CoA) reductase, a key step in the synthesis of cholesterol. The pleiotropic effects of statins may be connected with this basic mechanism. In this mechanism, not only the synthesis of cholesterol is reduced, but also that of the derivatives of mevalonic acid, including isoprenoids. These compounds participate in protein isoprenylation that links lipid fragments with intracellular proteins [23]. In limiting the production of isoprenoids, statins block the function of cytoplasmic regulatory proteins – GTP-ases from the *Rho* protein family, such as *Ras*, *Rac1* and *Rap*. This results in increased angiogenesis and myocardial perfusion, decreased myocardial apoptosis, and improvement in endothelial and cardiac function [24]. The deteriorating circulatory insufficiency is characterized by an increased amount of free radicals, which may inactivate nitric oxide (NO) [25,26]. Therefore, additional advantages of Rho protein inhibition are also connected with increased endothelial synthesis of NO and reduced expression of endothelin-1, which has a positive effect on endothelial function [25,26]. In addition, statins inhibit the synthesis of inflammatory cytokines and chemokines, improve autonomic function, and reverse myocardial remodeling [27,28].

Because of the pleiotropic effect of statins, there have been attempts to use these drugs in the treatment of DCM of nonischemic etiology. The present prospective, randomized study evaluated the effects of a small atorvastatin dose in 5-year observation on the parameters of inflammation, left ventricular function, hospitalizations and mortality in CHF patients with DCM who have already received standard HF therapy. In the atorvastatin group compared with the group without statin IL-6 and TNF-α concentrations were considerably lower. Also, UA concentration was lower in the atorvastatin group than in the group without statin therapy. No significant differences concerning NT-proBNP concentration, echocardiographic parameters of the left ventricle, distance in the 6-min walk test and in functional classification according to NYHA were observed between the examined groups. In the statin group after 5 years a decrease in NT-proBNP concentration compared with initial values and a decrease in LVdD and LVsD were achieved. LVEF significantly increased also in the atorvastatin group. Based on a comparison of curves using the log-rank test, the probability of survival to 5 years is higher in the group receiving a low dose of atorvastatin.

According to studies, hyperuricemia is an independent prognostic marker in chronic and in acute heart failure (AHF) [29,30]. Hyperuricemia can produce additional adverse effects on the cardiovascular system and can mediate the immune response [31,32]. Hyperuricemia in patients with heart failure is associated with higher levels of serum markers of inflammation (C-reactive protein [CRP], IL-6, and neutrophil count) [33] and higher levels of markers of endothelial activation, such as the soluble intercellular adhesion molecule-1, and inflammatory markers such as IL-6, TNF-α, and its receptors [34,35]. Although UA level has been associated with an increased risk of cardiovascular events, it is unclear whether UA can provide greater prognostic information than NT-proBNP in advanced HF with nonischemic DCM. In the study of Kim *et al.* UA and NT-proBNP values were obtained from 122 DCM patients. Development of clinical events during follow-up was defined as the composite of cardiac death and readmission for heart failure. During follow-up UA and NT-proBNP values were significantly higher in patients with events. On multivariate analysis, UA remained the only independent predictor of prognosis [35]. The authors’ findings demonstrated that UA value could be an informative predictor in nonischemic DCM. In our study we observed lower UA concentration in the group treated with atorvastatin, which perhaps may be connected with better prognosis in these patients. In DCM, an immunological component may play a role, so immunomodulatory effects of statins may be more advantageous. The study of Wojnicz *et al.*[36] evaluated the safety, tolerability, and efficacy of statin therapy in patients with heart failure secondary to inflammatory DCM and moderately elevated low-density lipoprotein cholesterol levels. Seventy-four patients were randomized to receive atorvastatin or conventional treatment for HF. After 6 months of therapy, the predefined primary efficacy end point (an increase of >5% in the absolute left ventricular ejection fraction and ≥2 selected criteria by echocardiography and a decrease in NYHA functional class) was significant in the statin-treated patients (*p* = 0.004). Among secondary efficacy parameters, the quality-of-life index showed a trend suggesting the benefit of statin therapy. These results suggest a positive anti-inflammatory effect of atorvastatin in patients with DCM. In the research of Gurguna *et al.*[37], the effectiveness of 12 weeks’ therapy with fluvastatin, 80 mg/day, was assessed concerning the concentration of inflammatory cytokines and LV function in patients with cardiac insufficiency and DCM as well as with cardiac insufficiency caused by coronary thrombosis. In both groups, a considerable improvement of ventricular function and clinical symptoms of cardiac insufficiency was achieved, as well as a decrease in the concentration of IL-6 and TNF-alpha [37]. In the study by Horwicha *et al.*[38], statin therapy was related to a higher survival rate without the necessity of urgent transplant in patients with cardiac insufficiency of ischemic origin as well as of non-ischaemic origin (91 *vs* 72%, *p* < 0.001 and 81 *vs* 63%, *p* < 0.001, respectively) [38]. Sola *et al.*[39] evaluated the influence of atorvastatin (20 mg/day) on vascular indicators of inflammation and echocardiographic indicators in 89 patients with dilated cardiomyopathy of nonischemic origin in NYHA class II to IV, with LVEF <35%. In the group treated with atorvastatin, considerable reduction of end diastolic and end systolic volume of the LV was obtained compared with the group treated with placebo [39]. In the statin group they observed higher LVEF and a considerable decrease in the concentration of hsCRP, TNF-α receptor 2, and IL-6, together with an increase of superoxide dismutase (E-SOD) activity in erythrocytes, which meant that oxidative stress and the inflammatory process decreased significantly within the 12-month observation. A significant improvement of clinical condition of patients in the atorvastatin group was also observed (NYHA class in this group 2.2 ± 0.3 compared with 2.9 ± 0.3 in the placebo group, *p* = 0.001) [39]. In the study by Node *et al.*[40], 53 patients with symptomatic DCM of nonischemic origin (NYHA class II and III) with LVEF <40% were assigned to a group receiving 10 mg of simvastatin or to a placebo group for 14 weeks. Patients treated with statin had considerably lower functional class according to NYHA and higher LVEF compared with patients from the placebo group. The concentrations of TNF-alpha, IL-6 and BNP were also significantly lower in the simvastatin group. The results of our study showing decreased IL-6 and TNF-α concentrations are in accord with Gurguna *et al.*[37], Horwich *et al.*[38], Sola *et al.*[39] and Node *et al.*[40]. We also observed a decrease in NT-proBNP concentration compared to initial values and a decrease in LVdD and LVsD in the group treated with atorvastatin.

On the other hand, Bleske *et al.*[41] randomly assigned 15 patients with DCM of nonischemic origin in functional class I to III according to NYHA to a group treated with 80 mg of atorvastatin or to a placebo group for 12 weeks. Although treatment was found to be safe and associated with considerable reduction of LDL cholesterol, the authors did not observe a significant difference between atorvastatin and placebo concerning NT-proBNP, hsCRP, TNF-alpha and indicators of endothelial activation: vascular adhesion molecule-1, intracellular adhesive molecule-1 and P-selectin [41]. In the study carried out by Krum *et al.*[42], the influence of rosuvastatin 40 mg in 86 patients with systolic heart failure (LVEF <40%) of ischemic or nonischemic etiology was assessed (68 patients with DCM). The primary end point was change in LVEF by radionuclide ventriculogram. Secondary end points included changes in echocardiographic parameters, neurohormonal and inflammatory markers, Packer composite score, death and HF hospitalization. Despite being safe and effective at decreasing plasma cholesterol, high-dose rosuvastatin did not beneficially alter parameters of LV remodeling [42].

In our study we observed better survival in the atorvastatin group of patients with DCM. The UNIVERSE and CORONA studies using rosuvastatin showed no beneficial effect on mortality in patients with mainly ischemic chronic HF [11,12]. In the *post-hoc* analysis of the Eplerenone Post-Acute Myocardial Infarction Heart Failure Efficacy and Survival Study (EPHESUS) [43], the initiation of statin therapy mainly during hospital stay for acute HF complicating acute myocardial infarction was associated with a lower risk of all-cause death. In a *post-hoc* analysis performed in 6632 patients included in the EPHESUS trial, 47% of patients had a statin prescribed at baseline. During a mean follow-up of 16 ± 7 months, all-cause death occurred in 12% of patients taking and in 18% of patients not taking a statin (*p* < 0.001). The risk of all-cause death was 20% lower in patients on statin. The reduction of all-cause death appears to be mainly attributable to a lower rate of cardiovascular death, especially sudden death and stroke [43].

The GISSI-HF trial [13] is the only large prospective study with some relevance to DCM because rosuvastatin was examined in a mixed population with heart failure. Rosuvastatin 10 mg/day did not affect clinical outcomes (death, hospitalization for cardiovascular reasons) in patients with CHF of any cause. However, the number of patients with DCM was small [13]. Treatment with rosuvastatin was safe [13].

To determine whether statin therapy improves survival in patients with heart failure secondary to nonischemic DCM, data from 1024 patients (NYHA functional class III and IV) with LVEF ≤0.35, who were enrolled in the BEST (Beta-blocker Evaluation of Survival Trial) trial were analyzed [44,45]. Statin therapy was independently associated with decreased all-cause mortality (HR 0.38, 95%CI 0.18–0.82, *p* = 0.0134) and cardiovascular death (HR 0.42, 95%CI 0.18–0.95, *p* = 0.037) [45]. Sudden deaths due to rapid ventricular arrhythmias account for ~ 50–80% of all deaths in patients with idiopathic DCM. This reduction of deaths might be caused, in part, by a reduction in arrhythmic sudden death [46,47]. Confirmation of this thesis can be found in the study by Xian-Zhi *et al.*[48], where early and intensive atorvastatin therapy significantly decreased the recurrence of ventricular premature beat or non-sustained ventricular tachycardia.

The study by Buber *et al.*[49] was performed in a subset of 821participants in the Multicenter Automatic Defibrillator Implantation Trial with Cardiac Resynchronization Therapy (MADIT-CRT) trial with a diagnosis of heart failure other than ischemic. In this analysis of data covering 821 patients, 499 of them received statins. Multivariate analysis showed that time-dependent statin therapy was independently associated with a significant 77% reduction in the risk of fast ventricular tachyarrhythmias (VT/VF) or death (*p* < 0.001) and with a significant 46% reduction in the risk of appropriate implantable cardioverter defibrillator shocks (*p* = 0.01). Consistent with these findings, the cumulative probability of fast VT/VF or death at 4 years of follow-up was significantly lower among patients who were treated with statins (11%) as compared with study patients who were not treated with statins (19%; *p* = 0.006 for the overall difference during follow-up). The study demonstrated that the use of statins is associated with a significant reduction in life-threatening arrhythmias in patients with nonischemic heart failure [49].

One of the potential explanations why recent prospective studies using hydrophilic rosuvastatin have not shown any beneficial effect on mortality [13,50-52] may be connected with the observation that metabolic and cardiac effects may differ between the lipophilic and hydrophilic statins. It may be suggested that one of the beneficial mechanisms of statins could be to rapidly affect signaling pathways in cell membranes of the myocardium and/or the autonomic nervous system, thereby protecting patients from life-threatening arrhythmias. This assumption would be in line with data showing statins to improve autonomic neural control and increase electrical stability of the myocardium [13,48-52]. The highly lipophilic statins such atorvastatin and simvastatin become easily embedded into the membrane, having overlapping locations in the hydrocarbon core adjacent to the phospholipid head groups [48]. Gao *et al.* reported that lipophilic simvastatin therapy in pacing-induced CHF inhibited NADPH oxidative activity in the rostral ventrolateral medulla and reduced the central sympathoexcitatory response in association with improvement in LV function [53]. Activation of the sympathetic nerve system is one of the important prognostic predictors for CHF patients [54]. Tsutamoto *et al.* randomized 63 stable outpatients with DCM to atorvastatin (n = 32) or rosuvastatin (n = 31) therapy. They evaluated cardiac sympathetic nerve activity by cardiac 123I-metaiodobenzylguanidine (MIBG) scintigraphy, hemodynamic parameters and neurohumoral factors before and after 6 months of treatment [55].

The level of plasma oxidized LDL (oxLDL), a biomarker of oxidative stress in the failing heart, is an independent prognostic predictor in CHF patients [56]. The clinical studies suggested that lipophilic statins improve cardiac sympathetic activity by reducing oxidative stress [57,58]. Mason *et al.* reported that the antioxidant effects of an active metabolite of atorvastatin were stronger than those of rosuvastatin [59]. Therefore, the increase in LVEF observed in the atorvastatin group may be partly related to an improvement of the oxidative stress in the myocardium. Li *et al.*[60] explored the effect of early statin therapy (atorvastatin and simvastatin) on mortality in patients with nonischemic DCM. A total of 315 patients with nonischemic DCM were enrolled. The median follow-up period was 45.1 months. By single-factor analysis, they found that the follow-up mortality was 17% in the statin group and it was significantly lower than the 37% mortality of non-statin users (*p* = 0.003); in patients with worsening cardiac function NYHA III-IV, the mortality of the statin group was 17% while a much higher mortality of 47% was found in non-statin users (*p* = 0.003). The authors concluded that early treatment with atorvastatin or simvastatin was closely correlated with the reduction of mortality in nonischemic dilated cardiomyopathy patients, which is consistent with our findings [60-62]. Our findings of better survival in the atorvastatin group are consistent with Vrtovec *et al.*[14], Domanski *et al.*[45] and Li *et al.*[60] and may support the underlying mechanism described by Buber *et al.*[49] and Tsutamoto *et al.*[55].

Our study has several limitations that include the relatively small number of patients at 5-year follow-up. The dose of statin after 2-month therapy in the atorvastatin group was adjusted individually to 10 or 20 mg. The open-trial methodology (not double-blinded study) need to be considered as a study limitation.

In conclusion, the pleiotropic effects of atorvastatin in a small dose (10–20 mg/day) significantly reduce levels of inflammatory cytokines (TNF-α, IL-6) and uric acid, as well as improve hemodynamic parameters (LVdD, LVsD and LVEF) in DCM patients after 5 years of treatment, and have a significant impact on the survival of this group of patients.

## Competing interests

The authors declared that they have no competing interests.

## Authors’ contributions

ABD planned the study protocol, took care about the patients and coordinate the research, DPM drafted the manuscript and revised it critically for important intellectual content, MR took part in the data analysis and drafted the manuscript, SvH took part in the data analysis and drafted the manuscript, JR drafted the manuscript, MB conceived of the study, participated in its design, and coordination, and prepared the final version of the manuscript. All authors read and approved the final manuscript.
